# Influence of the Expression of Inflammatory Markers on Kidney after Fetal Programming in an Experimental Model of Renal Failure

**DOI:** 10.1155/2016/9151607

**Published:** 2016-11-28

**Authors:** Carlos Donizete Pereira Júnior, Camila Souza de Oliveira Guimarães, Aline Cristina Souza da Silva, Aldo Rogelis Aquiles Rodrigues, Maria Aparecida da Glória, Vicente de Paula Antunes Teixeira, Niels Olsen Saraiva Câmara, Lenaldo Branco Rocha, Marlene Antônia dos Reis, Juliana Reis Machado, Laura Penna Rocha, Fernanda Rodrigues Helmo, Rosana Rosa Miranda Corrêa

**Affiliations:** ^1^Discipline of General Pathology, Institute of Biological and Natural Sciences, Federal University of Triângulo Mineiro, Uberaba, MG, Brazil; ^2^Department of Health Sciences, Lavras Federal University, Lavras, MG, Brazil; ^3^Discipline of Physiology, Institute of Biological and Natural Sciences, Federal University of Triângulo Mineiro, Uberaba, MG, Brazil; ^4^Nephrology Division, Federal University of São Paulo (UNIFESP), São Paulo, SP, Brazil; ^5^Department of Immunology, Institute of Biomedical Sciences IV, University of São Paulo (USP), São Paulo, SP, Brazil; ^6^Department of General Pathology, Institute of Tropical Pathology and Public Health, Federal University of Goiás, Goiânia, GO, Brazil

## Abstract

*Objective.* To evaluate the expression of inflammatory markers in experimental renal failure after fetal programming.* Methods.* The offspring aged two and five months were divided into four groups: CC (control dams, control offspring); DC (diabetic dams, control offspring); CFA (control dams, folic acid offspring, 250 mg/Kg); and DFA (diabetic dams, folic acid offspring). Gene expression of inflammatory markers MCP-1, IL-1, NOS3, TGF-*β*, TNF-*α*, and VEGF was evaluated by RT-PCR.* Results.* MCP-1 was increased in the CFA and DFA groups at two and five months of age, as well as in DC5 when compared to CC5. There was a higher expression of IL-1 in the CFA2, DFA2, and DC2 groups. There was a decrease in NOS3 and an increase in TNF-*α* in DFA5 in relation to CFA5. The gene expression of TGF-*β* increased in cases that had received folic acid at two and five months, and VEGF decreased in the CFA5 and DFA5 groups. DC5 showed increased VEGF expression in comparison with CC5.* Conclusions.* Gestational diabetes mellitus and folic acid both change the expression of inflammatory markers, thus demonstrating that the exposure to harmful agents in adulthood has a more severe impact in cases which underwent fetal reprogramming.

## 1. Introduction

The theory of fetal origins of adult disease (FOAD) proposed by Barker et al. (1986) claims that physiological changes during intrauterine development would promote the restriction of development, as well as ultrastructural and physiological changes that would predispose to the early development of cardiovascular and metabolic diseases in adulthood [1–3].

In a model of diabetes in pregnancy, it was demonstrated that hyperglycemia promotes oxidative stress in the offspring, hence affecting the balance between oxidant and antioxidant factors [4], an increase in inflammatory markers, and a reduction in the number of glomeruli with aging [5].

The Streptozotocin (STZ) may cause mild or severe diabetes mellitus (DM) in the dam and lead to different effects in rat offspring [6]. Early blood pressure, deficit in glomerular filtration and in renal plasma flow, and also glomerular hypertrophy were observed as the offspring aged [5].

Acute renal failure (ARF) is a kidney disorder which may result from reduced renal perfusion with no cell injury; from ischemic, toxic, or obstructive injury of the renal tubules; from tubulointerstitial inflammation and swelling; or from reduced glomerular filtration rate associated with primary glomerular diseases [7].

The literature shows the relationship between pathological changes in adulthood and changes during intrauterine development, particularly DM. Thus, it is believed that renal injuries in a fetal programming model are caused by persistent renal inflammatory response due to an injury during intrauterine life [8].

Some authors demonstrated that gestational diabetes mellitus may lead to modifications in the placental transcriptome characterized by dominance of genes that regulate inflammatory responses [9]. Cytokines play a crucial role in the establishment of inflammatory response and the systemic inflammation is associated with the development of endothelial dysfunction and hypertension which may result in progression of chronic disease [10]. As inflammatory markers may predict later metabolic [11, 12] and vascular [13] disease, it is extremely important to measure inflammatory markers in a fetal programming model.

Therefore, based on the change of the current profile of patients with ARF and on the association of this entity with several chronic diseases, the aim of this study was to evaluate the progression of folic acid-induced ARF in a fetal programming model through the expression of proinflammatory markers.

This study aimed to demonstrate the influence of fetal programming on the development of ARF, as well as the prognostic evolution of individuals that develop the disease.

## 2. Methods

This study was submitted to the Ethics Committee on Animal Use of the Federal University of Triângulo Mineiro and approved under protocol number 168.

Male and female Wistar rats, with initial weight ranging from 250 to 330 g, were maintained on a 12 h light-dark cycle at a constant temperature (25°C).

### 2.1. Diabetes-Induction Model

**Figure 1:**
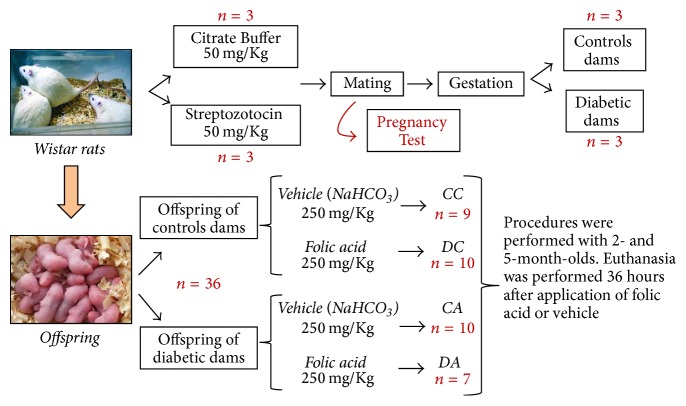
Steps of the experiment.

DM was induced by STZ at a dose of 50 mg/Kg after twelve hours of fasting; it was administered intraperitoneally at a single dose into female rats weighing 250–330 g. Control animals received the same dose of vehicle (0.1 M Citrate Buffer, pH 4.5). The diabetic state was confirmed after 48 hours by blood glucose measurement. Only animals with blood glucose levels above 250 mg/dL were considered dams (Figure 1).

### 2.2. Mating

After the induction of diabetes, the estrous cycle of each rat was determined according to criteria established in the literature [14]. After a regular cycle, the diabetic rats were mated at a ratio of one male to one female, and then a pregnancy test was performed through microscopic examination of vaginal secretions. The period between the establishment of the diabetic state and mating lasted 3–7 days. Females that had spent a period of over seven days and/or two regular cycles with a male but that did not get pregnant were considered infertile and discarded in this study (Figure 1).

### 2.3. Selection of Animals

Approximately 21 days after birth, all the offspring from each dam were kept in lactation. In this stage, the pups stayed with the mother in a single cage. From the 28th day of life, the male rats were separated from their mothers and placed in collective cages. Male animals at two and five months of age were studied, comprising a total of 36 young males (Figure 1).

### 2.4. Development of ARF

**Figure 2:**
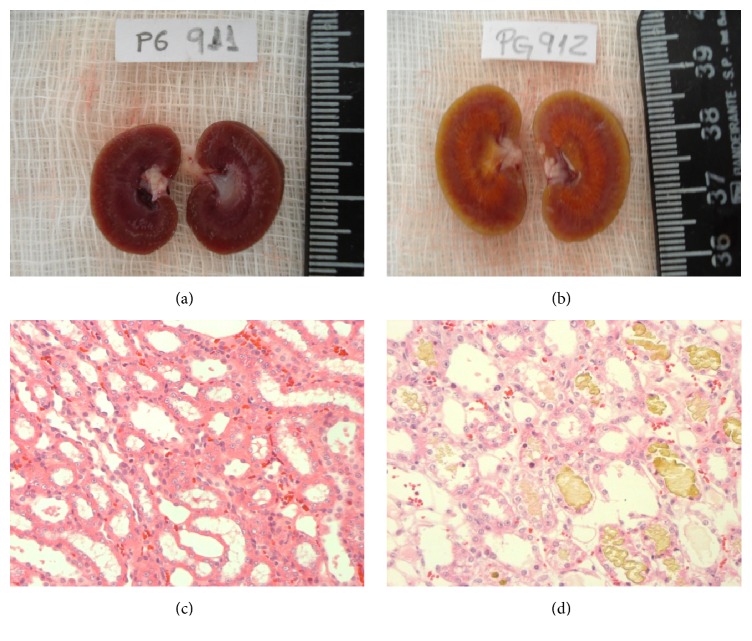
Development of acute renal failure. (a) Normal kidney of offspring who received vehicle; (b) kidney of offspring who received a single intraperitoneal injection of folic acid, characterized by a yellowish color and increased size; (c) normal kidney of offspring who received vehicle; (d) kidney of offspring who received a single intraperitoneal injection of folic acid, which present inside the tubules a granular and yellowish material compatible with folic acid.

The following experiments were performed when the animals reached the age of two and five months: the folic acid group received a single intraperitoneal injection of folic acid (Sigma, St. Louis, MO) at a dose of 250 mg/kg in vehicle (0.2 mL of 300 mM NaHCO3); and the same volume of vehicle was administered to control animals (Figures 1 and 2).

### 2.5. Groups

The animals were divided into four groups, as follows: (1) CC: control dam and control offspring; (2) CFA: control dam and folic acid offspring; (3) DC: diabetic dam and control offspring; and (4) DFA: diabetic dam and folic acid offspring.

### 2.6. Euthanasia

Euthanasia was performed 36 hours after administration of folic acid. The animals were anaesthetized with a single injection of Ketamine (60 mg/kg IM) and Xylazine (5 mg/Kg IM), followed by exsanguination.

### 2.7. Collection of Sections

Both kidneys of each animal were collected, weighed, and photographed, and then the renal capsule was sectioned and removed. The right kidney was used for histological analysis and the left kidney was used for molecular biology analysis of gene transcripts. Half of the left kidney was divided into sections of approximately 50 mg, placed in a 1.5 mL polystyrene tube (Eppendorf®, Hamburg, Germany), and immediately frozen in liquid nitrogen and transferred to a freezer at –80°C; the other half was fixed in Karnovsky's fixative for electron microscopy and in isopentane for further analysis.

### 2.8. Real-Time Polymerase Chain Reaction (RT-PCR)

Gene expression of monocyte chemoattractant protein-1 (MCP-1), interleukin-1 (IL-1), NOS3, transforming growth factor-*β* (TGF-*β*), tumor necrosis factor-*α* (TNF-*α*), and vascular endothelial growth factor (VEGF) was measured by using TaqMan® amplification system (Applied Biosystems®, Branchburg, New Jersey, USA), consisting of a commercial reagent mixture (TaqMan Universal PCR Master Mix, Applied Biosystems) and of specific custom primers and probes (Assay-on-Demand® 20x, Applied Biosystems). The reactions using TaqMan system consisted of the following volumes: Mix TaqMan (5 *μ*L), primer/probe (0.5 *μ*L), cDNA (1 *μ*L), and sterile water* q.s.* 10 *μ*L.

#### 2.8.1. Ribonucleic Acid (RNA) Extraction

(1) Less than 100 mg of renal tissue was triturated in 1 mL of Trizol® (Invitrogen®, Carlsbad, California, USA) using a Polytron PT 1200 CL® homogenizer (Kinematica AG®, Littau, Switzerland); (2) the homogenate was incubated for five minutes at room temperature, and 200 *μ*L of chloroform p.a. was added (Merck®, Darmstadt, Germany); (3) the homogenate was incubated for three minutes and centrifuged at 12,000 ×g at 4°C for 15 minutes; the mixture formed three distinct phases: an organic phase containing proteins, a white interphase (DNA precipitate), and a colorless aqueous phase containing total RNA; (4) the aqueous phase was transferred to a new tube, to which 500 *μ*L isopropanol p.a. (Merck) was added, thus promoting the precipitation of RNA; (5) the sample was incubated for ten minutes and then centrifuged for 10 minutes. The precipitated RNA formed a substance of gelatinous consistency; the supernatant was removed and 1 mL of 75% p.a. ethanol solution (Merck) was added to remove the salts from the Trizol reagent and then homogenized in a vortex mixer; (6) this solution was centrifuged at 10,500 ×g in a refrigerated centrifuge for five minutes; (7) the supernatant was removed, and the centrifuged solution was dried for ten minutes and was then rediluted in 50 *μ*L of RNase/DNase-free water; (8) the RNA was stored in a freezer at –80°C.

#### 2.8.2. Synthesis of Complementary Deoxyribonucleic Acid (cDNA)

It was synthesized from 2 *μ*g of total RNA previously treated with DNase (RQ1 RNAse-free DNase) (Promega®, Madison, USA). The following were added to the treated RNA: 0.2 *μ*g of Oligo(dT)12-18 (GE Healthcare®, Buckinghamshire, UK), 20 ng of BSA (Bovine Serum Albumin), 0.1 *μ*mol of dNTPs (Promega), and 400 U of M-MLV reverse transcriptase enzyme (Promega). This mixture (50 mL) was incubated at 37°C for one hour for reverse transcription reactions. All samples were run in triplicate containing a final reaction volume of 10 *μ*L, as follows: 5 *μ*L of TaqMan Universal PCR Master Mix; 1 *μ*L of cDNA from each sample; 0,5 *μ*L of primer and probe mix; and, in the end, ultrapure water* q.s.* 10 *μ*L. The reaction was performed in a 7500 Real-Time PCR System® thermal cycler (Applied Biosystems, Singapore). Amplification conditions for the TaqMan system are standardized and universal for any amplified PCR product. A comparative analysis of the cycle thresholds (CT) was used in order to determine the gene expression of MCP-1 (Rn00580555_m1), IL-1 (Rn00580432_m1), NOS3 (Rn02132634_s1), TGF-*β* (Rn00572010_m1), TNF-*α* (Rn99999017_m1), and VEGF (Rn01511601_m1). For each sample, the CT values of the target genes were normalized by their respective control gene, and the value used to demonstrate the relative expression of target genes was determined using the 2^−ΔΔCT^ expression. Thus, relative mRNA levels were expressed as a difference of “*n*” times regarding a control sample; in this study, the same sample was always used at baseline.

### 2.9. Statistical Analysis

Normality was assessed using the Kolmogorov-Smirnov test. For analysis between groups, One Way ANOVA (*F*) followed by Tukey test was used. The results were expressed as mean ± standard deviation (*X* ± SD). Values of *p* < 0.05 were considered statistically significant.

## 3. Results

**Figure 3:**
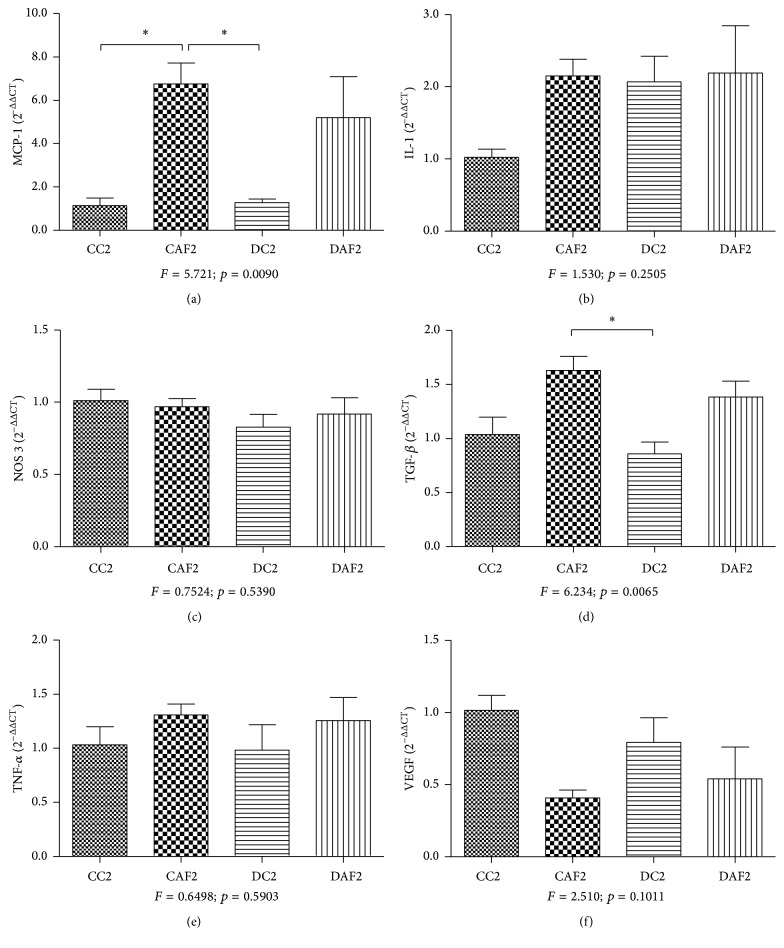
mRNA gene expression of MCP-1, IL-1, NOS3, TGF-*β*, TNF-*α*, and VEGF in the kidney of control offspring and diabetic offspring with or without folic acid, aged two months. (a) The gene expression of MCP-1 was increased in the CFA2 group; (b and c) the gene expression of IL-1 and NOS3 showed no significant difference between the study groups; (d) the gene expression of TGF-*β* was decreased in the DC2 group; (e and f) the gene expression of TNF-*α* and VEGF showed no significant difference between the groups. ^*∗*^Significant differences.

**Figure 4:**
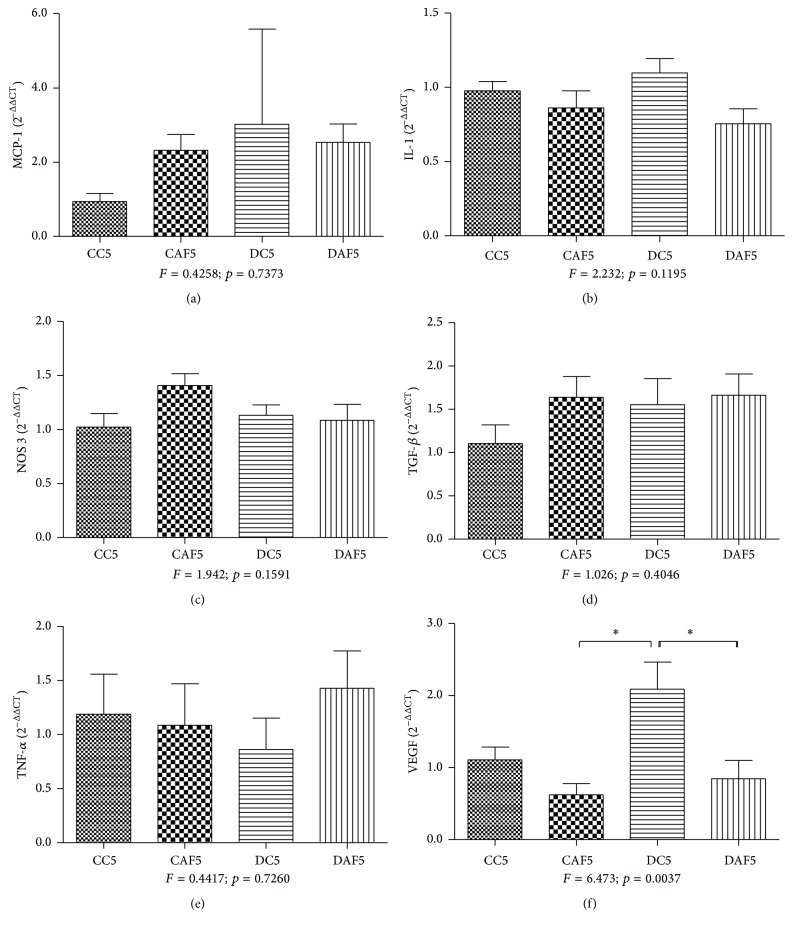
mRNA gene expression of MCP-1, IL-1, NOS3, TGF-*β*, TNF-*α*, and VEGF in the kidney of control offspring and diabetic offspring with or without folic acid, aged five months. (a, b, c, d, and e) The gene expression of MCP-1, IL-1, NOS3, TGF-*β*, and TNF-*α* showed no significant difference between the study groups; (f) the gene expression of VEGF was decreased in the CFA5 and DFA5 groups. ^*∗*^Significant differences.

RT-PCR analysis showed an increase in gene expression for MCP-1 in the groups that had received folic acid (CFA2 and DFA2) in comparison with their respective control groups. The CFA2 group presented MCP-1 significantly higher than the groups CC2 and DC2 (Figure 3(a)). MCP-1 expression was similar in animals aged five months that had been treated with folic acid. This expression was higher in DC5 cases than in CC5 cases, however, without significant difference between groups (Figure 4(a)).

There was a higher gene expression for IL-1 in CFA2, DFA2, and DC2 cases in relation to CC2 cases, however, without significant difference between groups (Figure 3(b)). Regarding the animals aged five months, IL-1 expression was similar among the four groups analyzed (Figure 4(b)).

Gene expression for NOS3 showed no significant difference in expression between the groups (Figures 3(c) and 4(c)). On the other hand, NOS3 was found to be reduced in the DFA5 group in comparison with the CFA5 group, despite the absence of a significant difference (Figure 4(c)).

Nonetheless, a significant increase in gene expression of TGF-*β* was observed between the groups that had received folic acid (CFA2 and DFA2) when compared to their respective control groups. The CFA2 group presented TGF-*β* significantly higher than DC2 group (Figure 3(d)). There was no significant difference in the expression of TGF-*β* between the groups analyzed at five months. Nevertheless, DC5 cases and the offspring of control and diabetic dams that had received folic acid had a higher expression of TGF-*β* than CC5 cases (Figure 4(d)).

The gene expression of TNF-*α* was increased in the cases that had received folic acid (CFA2 and DFA2) compared to their respective control groups, despite no significant difference (Figure 3(e)). There was also no significant difference in TNF-*α* expression between the cases analyzed at the age of five months, even though a higher expression was observed in the DFA5 group in comparison with the DC5 and CFA5 groups. The DC5 group had higher expression than the control group (Figure 4(e)).

On the other hand, the gene expression of VEGF was reduced in the CFA2 and DFA2 groups in relation to their respective control groups, however, without significant difference between groups (Figure 3(f)). Furthermore, there was a significant reduction of VEGF in the groups that had received folic acid (CFA5 and DFA5). The DC5 group showed a higher expression of VEGF than the CC5 group, despite no significant difference (Figure 4(f)).

## 4. Discussion

In this study, folic acid increased the expression of MCP-1 in the control offspring and in the diabetic offspring only in two-month group. In different experimental models, folic acid induces acute renal failure by tubular injury, which is characterized by apoptosis, proliferation of tubular cells, inflammatory infiltrate, mild fibrosis, podocyte edema, thinning of pedicel membrane associated with the presence of vesicles, reduced brush border, and tubular hypertrophy and obstruction [15] due to the deposition of folic acid crystals. Therefore, MCP-1 seems to behave as an important biomarker of renal injury in the evaluated groups.

These changes, associated with hyperglycemia, are responsible for the increase in MCP-1. An experimental study on the offspring of diabetic rats correlated the expression of this proinflammatory cytokine with the chronic activation of the innate immune system in response to the increased insulin resistance [16]. Moreover, because of fetal programming, it is believed that the dysfunction of fetal pancreatic *β* cells caused by gestational DM results in permanent metabolic disturbance in postnatal and adult life [16, 17], severe insulin resistance [5, 16], a reduction in the number of nephrons and glomeruli, and impaired glomerular filtration rate [5]. Therefore, these data may explain the higher expression of MCP-1 in DC5 cases compared to the CC5 cases.

The expression of IL-1 was higher in the two-month groups, CFA2, DFA2, and DC2. IL-1 is a cytokine involved in different inflammatory diseases. This cytokine may mediate acute forms of renal injury, as well as being involved in the development of chronic kidney disease [18]. Thus, it can be inferred that the injuries caused by folic acid [15] and by fetal reprogramming [16] increase the expression of IL-1 in the two-month-old offspring.

An experimental study showed that intrauterine hyperglycemia causes the reduction of pancreatic islets, as well as a significant increase in glucose intolerance in the diabetic offspring from the 16th week of development [3]. These changes are correlated to the action of reactive oxygen species on pancreatic *β* cells, which activate the transmembrane Toll-like receptors responsible for the synthesis of IL-1 and of other cytokines in response to the cytotoxic effect [19]. Accordingly, fetal programming may led to a higher expression of IL-1 in DC progeny than in CC progeny, as we observed in the two-month-old offspring.

Comparing the two groups that suffered renal injury by folic acid, a decreased NOS3 in cases which suffered fetal reprogramming only in five-month groups was observed. NOS3 is expressed in the vascular endothelium of the afferent and efferent arterioles in the kidney [20]. However, in cases of renal injury, there is a severe uncoupling of NOS3 via the NADPH oxidase enzyme system and it is responsible for endothelial ROS production [21]. Thus, it can be inferred that oxidative stress interferes with its synthesis and activity. DM compromises the activity of phosphatidylinositol 3-kinase and the activation of protein kinase B, which is responsible for phosphorylation and activation of NOS3. Interference in this pathway inhibits the oxidation of L-arginine to NO as well as the stability of NOS3 mRNA [20]. The reduced expression of DFA5 compared to CFA5 also indicates a greater impact of folic acid injury on the diabetic progeny, and it shows that fetal reprogramming interferes with the development of changes.

Gene expression of TGF-*β* was higher in the CFA2 and DFA2 groups. This data is in accordance with the literature, since folic acid is associated with mitochondrial dysfunction in renal failure. An experimental study showed that TGF-*β* levels increase up to four times six days after induction of renal injury [22].

Therefore, the higher mRNA synthesis of this cytokine in podocytes contributes to mesangial matrix expansion, GBM thickening, and proliferation, hypertrophy, and apoptosis [23–25] of podocytes and proximal tubular cells. Thus, these findings seem to explain the higher expression of this cytokine in the CFA2 and DFA2 groups.

TGF-*β* gene expression also showed the influence of age on the analyzed progeny, since the DC5 group was older. The hyperglycemic state promotes glycation of proteins, diacylglycerol synthesis, and activation of protein kinase C that favor its expression [23, 26]. Hence, these results confirm the interference of fetal programming in the analyzed progeny. Moreover, the higher expression in the DC5 group compared to CC5 group indicates a more evident late renal failure.

TNF-*α* expression was higher in both the CFA2 and DFA2 groups and also in the DFA5 group when compared to the DC5 and CFA5 groups. Studies have showed that the levels of this inflammatory cytokine are increased by 25 times in the renal tissue of rats 48 hours after administration of folic acid, which was associated not only with a higher prevalence of epithelial apoptosis and necrosis [27, 28], but also with glomerular hypercellularity and mesangial expansion [29].

The literature shows that the development of maternal insulin resistance appears to be responsible for the increase in TNF-*α* levels from the first stage of prenatal development to the 28th week [30]. The increased expression of TNF-*α* in DFA5 cases in relation to CFA5 cases also indicates a greater impact of folic acid injury in diabetic offspring, hence indicating that fetal reprogramming interferes with the progressive changes that appear in late adulthood.

VEGF gene expression was lower in both the CFA2 and DFA2 groups, as well as in the CFA5 and DFA5 groups. Folic acid appears to interfere with VEGF expression, since there is a reduction in the expression of this growth factor 14 days after administration. The progression of nephrotoxicity is believed to be responsible for both attenuating receptor expression and for interfering with VEGF synthesis, which occurs via intracellular signaling pathways [31].

On the other hand, gene expression increased in the DC5 group. In DM, renal hypoxia promotes VEGF synthesis via the accumulation of hypoxia-inducible factor. Similarly, hyperglycemia caused by activation of extracellular signal-regulated kinase in podocytes and by advanced glycation end products (AGEs) [32] is responsible for the expression of this factor.

DM causes early ultrastructural changes, such as mesangial and endothelial cell proliferation, GBM thickening, and erasure of pedicels [33], as well as late ultrastructural changes, such as apoptosis of renal cells through increased expression of proapoptotic proteins Bim and Bax, and consequent increase in the activation of caspase-3 [34]. Therefore, the progression of this disease may have promoted gene expression of VEGF in diabetic offspring aged two and five months in this study.

Studies on fetal reprogramming in humans are scarce due to the difficulty of selecting adults who have suffered some type of aggression during their fetal development, which may be confirmed through maternal medical records and, therefore, be able to assess the late consequences of these aggressions. In the literature are found some longitudinal [35] and cohort studies [36] on human fetal reprogramming. There are also some studies with Holocaust survivors born during World War II proving the late effects of nutritional deficiency during fetal development on adults and their offspring due to maternal malnutrition and hunger. The results of these studies indicate that these individuals were more likely to present dyslipidemia, hypertension, vascular disease, diabetes mellitus, metabolic syndrome, and premature osteopenia/osteoporosis [37–39].

However, acquiring human samples of biopsies and associating the data with intrauterine aggression history may be very difficult or even infeasible. Because of this, experimental studies on fetal reprogramming are extremely important to simulate something that may occur in humans and, thus, be able to understand the molecular mechanisms involved in this process. This experimental study suggests that metabolic dysfunctions triggered by DM during pregnancy and by folic acid during intrauterine life culminate in fetal programming of descendant offspring in postnatal life. The decrease in NOS3 and increase in TNF-*α* indicate a greater impact of folic acid injury in diabetic offspring, and they also show that fetal reprogramming interferes with the development of changes that appear in late adulthood.
